# The disabling consequences of Mycetoma

**DOI:** 10.1371/journal.pntd.0007019

**Published:** 2018-12-10

**Authors:** Mustafa Abbas, Peter Siordet Scolding, Abubaker Ahmed Yosif, Roa Fath EL Rahman, Melka O. EL-Amin, Mohamed Kamal Elbashir, Nora Groce, Ahmed Hassan Fahal

**Affiliations:** 1 The Mycetoma Research Centre, University of Khartoum, Khartoum, Sudan; 2 Leonard Cheshire Research Centre, University College London, London, United Kingdom; University of California San Diego School of Medicine, UNITED STATES

## Abstract

Mycetoma is a neglected tropical disease endemic in tropical and subtropical countries, particularly Sudan. The disease is characterised by the triad of painless subcutaneous mass, multiple sinuses and discharge that contain grains. It is a chronic, debilitating disease most commonly affecting the feet or hands and leads to substantial morbidity, loss of function and even amputation. It predominantly affects poor, rural populations and patients typically present late with advanced disease and complications. In this descriptive cross-sectional study, we characterise the disabling consequences of mycetoma. The study included 300 patients; 228 (76%) male and 72 (24%) female with confirmed mycetoma seen at the Mycetoma Research Centre, University of Khartoum, Sudan in the period May 2016 and January 2017. The study design was based upon the International Classification of Functioning, Disability and Health, examining the impact of mycetoma on eight life domains. Our major finding is that mycetoma is a significantly disabling disease. Over 60% of the study population (181 patients) had moderate impairment or difficulty in at least one domain variable. The important disability was mobility impairment and walking difficulty that was reported in 119 patients (39.7%). There was significant pain associated with mycetoma lesions in 103 patients (34%), challenging the traditional view of mycetoma as a painless disease. The economic burden was also found to be substantial, with 126 patients (46.7%) reporting barriers to their ability to sustain themselves. This is the first study evaluating the disabling consequences of mycetoma and shows clear areas for intervention and further research. Options for mitigating social and economic impacts include routine integration of analgesia and physiotherapy into treatment protocols, and adapting educational provision and working practices based on disability assessment. Our data show that mycetoma is a public health issue with direct implications on quality of life.

## Author summary

Mycetoma is a neglected tropical disease endemic in many tropical and subtropical countries affecting poor, rural populations. It commonly affects the feet or hands and leads to substantial chronic morbidity, loss of function and disability. The disabling consequences of mycetoma have not been studied before. In this study we interviewed 300 patients and asked them to what extent mycetoma has affected their lives across several functional domains. We looked at bodily function, mobility, self-care, domestic activities, interpersonal interactions and relationships, education, economic status, and community, social and civic life. We found that mycetoma causes significant disability and pain. It also causes a significant financial burden to patients, interfering with their ability to be economically self-sufficient and to gain remunerative employment. We also found that mycetoma prevents the attainment of education. To overcome mycetoma-related disability we recommend integrating analgesia and physiotherapy into clinical services, adapting educational provision and working practices and early case detection and treatment to avoid the disease disabling consequences.

## Introduction

Mycetoma is a neglected tropical disease frequently affecting the poorest of the poor in remote, poor communities [1]. It is endemic in many tropical and subtropical countries around the world including Sudan [2, 3]. The geographical distribution of mycetoma depends on a range of environmental factors such as rainfall, humidity and temperature [4, 5]. Mycetoma has a higher prevalence in rural areas with poor domestic hygiene [6, 7]. The disease is caused by more than 50 microorganisms of fungal or bacterial origin, and hence it is classified as eumycetoma and actinomycetoma respectively [8, 9]. Mycetoma is believed to occur as a result of traumatic implantation of the causative organism into the subcutaneous tissue via minor trauma or injury [10, 11]. It then spreads to involve the skin, deep tissues and bone leading to massive destruction, deformities and disabilities [12, 13]. If untreated it can have a major impact on the affected patients, communities and the health system in endemic countries [14, 15].

The disease is characterised by the triad of subcutaneous mass, multiple sinuses and discharge containing grains [16, 17]. It is most frequently seen in the foot and hand, which account for 86% of reported cases, though no site is exempt [18, 19]. Mycetoma is most prevalent amongst children and young adults, with patients frequently presenting with advanced disease. Delayed presentation may result from patients’ low socio-economic status and health education levels, and the paucity of health services in distant, isolated endemic areas. Overall, these factors lead to a devastating socio-economic impact [20, 21, 22, 23, 24]. Currently, the available treatments (combination antimicrobial therapy for actinomycetoma, or antifungal therapy for eumycetoma) have proved ineffective and expensive, with a range of side effects and high recurrence rate [25, 26]. Late presentations often necessitate amputation or destructive surgical excisions [27, 28, 29]. Patients require long-term follow-up to monitor recovery and recurrence, and unfortunately these factors as well as travel and opportunity cost of attending clinic lead to a high loss to follow up.

The disabling consequences of mycetoma are poorly understood. A medical literature search revealed no report addressing the present study objectives, and hence this study was designed to determine the disabling impact of mycetoma upon patients. We aim to identify key areas for future study with the ultimate goal of building evidence-based intervention and appropriate health care for those affected.

The mycetoma-related disability burden is hypothesised to be significant for several reasons. Firstly, the disease leads to structural impairment of the limbs, the most commonly affected parts, and disability arises from the direct loss of function. Secondly, current treatment options are limited and suboptimal, with disease potentially lasting for decades, resulting in increasing impairments over time. Finally, support and adaptation for individuals with any disabling consequences of mycetoma are limited in Sudan, as indeed they are for many other disabling neglected conditions around the world.

As far as the authors know, this is the first-ever study of the disabling consequences of mycetoma.

## Material & methods

### Study design

The study was designed using the International Classification of Functioning, Disability and Health (ICF). The ICF is a standardized framework and classification for the description of health and health related states [30]. It conceptualises disability as an interaction between the patient health condition and his/her environment. Functions relate to body functioning (emotional state, pain, physical movement etc.), activities (driving, walking, etc.), and participation in society (engaging with religious ceremonies, etc.). Disability in this framework is a term for impairments or restrictions in functioning [31].

Study design, questions and qualifiers were taken directly from the ICF. Questions were selected based on their relevance to mycetoma and were purposefully broad, encompassing a range of possible impacts. All the possible questions in the ICF were reviewed; six questions from Body Functioning and 27 from Activities and Participation, thus 33 variables were selected. Answers to the questions were not ‘yes’ or ‘no’. Rather, grades of severity of the impairment or difficulty with a scale of mild, moderate, severe, or complete were used to further delineate the impact of the disease on individuals. These qualifiers for each grade were taken from the ICF (Table 1).

**Table 1:** Description of qualifiers.

Qualifier	Description	Score Given
No impairment or difficulty	A person has no problem	0
Mild impairment or difficulty	A problem that is present less than 25% of the time, with an intensity which a person can tolerate and which happens occasionally over the last 30 days	1
Moderate impairment or difficulty	A problem that is present less than 50% of the time, with an intensity which is interfering in a person’s day to day life and which happens occasionally over the last 30 days	2
Severe impairment or difficulty	A problem that is present more than 50% of the time, with an intensity which is partially disrupting the person’s day to day life and which happens frequently over the last 30 days	3
Complete impairment or difficulty	A problem that is present more than 95% of the time, with an intensity which is totally disrupting to the person’s day to day life and which happens every day over the last 30 days	4

### Data collection

The data was collected by four recently graduated doctors. Prior to the data collection, they had a four-day training course on data collection and interview skills provided by the first author. The training also included role-play, real-life interview assessment and an examination at the end of the course, following which their first two patients’ interviews were additionally observed for performance quality. They each passed the different parts of the training course.

The interviews were conducted each Monday at the Mycetoma Clinic of the Mycetoma Research Centre (MRC), University of Khartoum, between May 2016 and January 2017. To prevent selection bias, patients with confirmed mycetoma were given a number on arrival. Every third patient was chosen to be part of the study. Patients attended the clinic for their regular appointment and were interviewed either before or after their review. Patients who attended clinic Tuesday-Friday were not interviewed. We were able to survey 300 patients within the defined time scale of the project. Patients were given the option to decline and the interview was conducted in privacy. Ethical clearance was obtained from the Mycetoma Research Centre IRB and every patient gave verbal informed consent.

### Data analysis

A scoring system for the various mycetoma-related disability variables studied was created. The variables included impairment in bodily functions or difficulty in activities and participation in society. A mild impairment or difficulty was given a value of one; that of moderate was given a value of two; severe was given a value of three, and complete inability to perform a given task or participate in activity was given a value of four. These values were added up over the 33 variables into a cumulative disability score that was used during data analysis. The statistical software used was Statistical Package for the Social Sciences and a P value of <0.05 denoted statistical significance. Correlations were determined using a Pearson co-efficient bivariate two-tailed test.

## Results

### Demographic

Between May 2016 and January 2017, 300 patients were interviewed; of these 228 (76%) were males and 72 (24%) were females with a gender ratio of 3:1. The mean age of the studied participants was 32.1 years with a sample standard deviation of 14.0 years; the youngest was 5 years old and the oldest was 74 years old; 39% of them were between the ages of 5–25 years, 49% between 26–50 years, and 12% were aged 51 to 74 years. The participants were most commonly students (16%), farmers (13%), and housewives (11.3%). In 36% of participants, the occupation was unspecified.

The mycetoma mean duration was 7.8 years with a sample standard deviation of 6.6 years and a range of three months to 34 years. 50% of participants had had mycetoma for 0–5 years, 23% for 5–10 years, and 27% for more than 10 years. Most individuals (270, 90%) reported a mycetoma lesion in the lower limb (hip through to toes), 23 patients (8%) had a mycetoma lesion in the upper limb, six patients (2%) reported the lesion in the head and neck, and one participant reported mycetoma in multiple sites. Most patients (203, 68%) had had local surgical excision (most frequently at rural hospital sites), of these 140 (69%) had had recurrence. Seven patients had undergone amputation, of whom two had had recurrence.

### Ethics statement

Ethical clearance was obtained from the Mycetoma Research Centre IRB and every patient gave verbal informed consent. Written consent was decided to be not necessary as the patients’ identity was not disclosed in this article. The patients’ verbal informed consent acceptance was documented in each questionnaire during the interview.

### Cumulative disability scores

The study showed that 49 (16.3%) of all participants had no difficulty or impairment in any of the 33 studied variables (Table 2). These patients were slightly younger, with a mean age of 29 years and an age range of 7 to 65; and they had had mycetoma for a shorter duration than the average (mean 6.9 years, range 0.5–20 years). Their most common occupations were students (22%) or housewives (16%). All other patients (251, 83.7%) experienced disability ranging from mild to severe. The mean cumulative disability score for all degrees of disability among the studied patients was 10.7 with a range of 0 to 68. Most of the patients (133, 44.3%) had a score between 1 and 10. 62 patients (20.7%) had a score of 10–20, 31 patients (10.3%) had a score of 20–30, and 25 patients (8.3%) had a score of more than 30. The study showed that 181 patients (60.3%) had moderate impairment or difficulty in at least one domain variable. The scores were highest among farmers, those not in income-generating work, elderly patients and those with a longer disease duration.

**Table 2:** Overall assessment of mycetoma disability using cumulative disability scoring.

Disability Scoring		No.	%
All patients	No difficulty/Impairment	49	16.3
	Score of 1–10	133	44.3
	Score of 10–20	62	20.7
	Score of 20–30	31	10.3
	Above 30	25	8.3
	Total	300	100.0

### Results by variable

**Fig 1:**
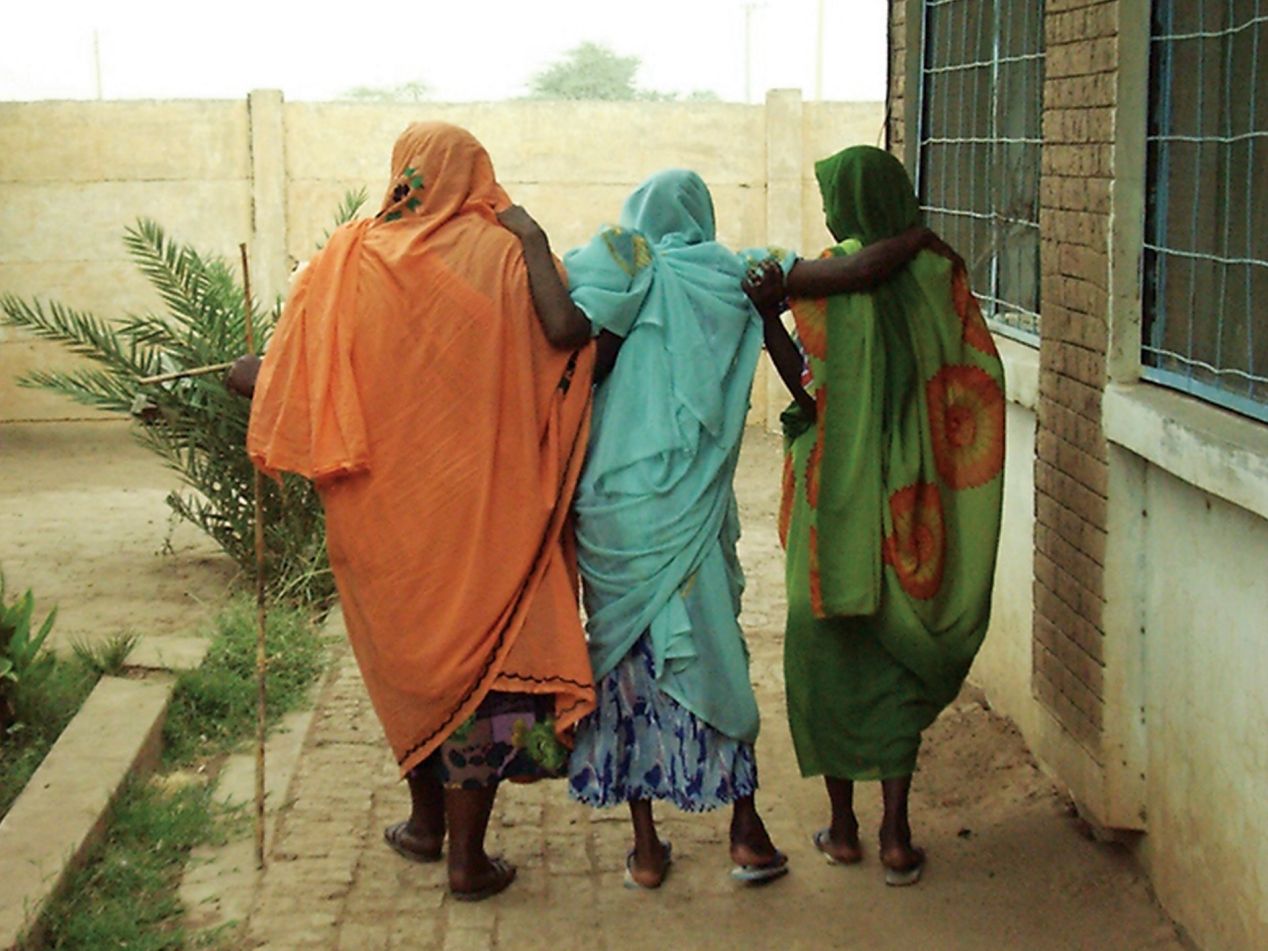
A lady with mycetoma supported by two family members to execute her normal live activities.

In this study, 103 patients (34.3%) had local pain of various degrees at the mycetoma site (Table 3). Mild pain was confirmed by 70 patients (23.3%), 23 patients (8%) had moderate pain, and 10 (3%) had severe pain. Worsening of pain was statistically significantly correlated with advanced age; (*p*<0.01). No results are available for analgesic control in this population (Fig 1).

**Table 3:** Impairments in body function.

Degree of Impairment	Energy and Drive	Sleep	Emotional	Body Image	Pain Sensation	Weight Maintenance
None	220	269	224	216	197	236
Mild	52	18	47	37	70	46
Moderate	26	11	28	42	23	17
Severe	01	02	01	03	10	01
Complete	00	00	00	00	00	00
Missing records	01	00	00	02	00	00
Total no. with some impairment	79	31	76	82	103	64
% with impairment	26.4	10.3	25.3	27.5	34.3	21.3

In this study, 119 patients (39.7%) reported walking difficulty, (Table 4): 54 patients (18%) had mild difficulty, 36 patients (12%) had moderate difficulty, 23 patients (8%) had severe difficulty, and six patients (2%) were completely unable to walk. Worsening of walking ability was statistically significantly associated with advanced age (*p*<0.01), longer disease duration (*p*<0.01), and pain at the lesion site (*p*<0.01).

**Table 4:** Difficulties in mobility.

Capacity	Hand & Arm Use	Walking	Transportation	Driving
No difficulty	273	181	214	93
Mild Difficulty	19	54	47	09
Moderate Difficulty	05	36	19	01
Severe Difficulty	01	23	09	04
Complete Inability	01	06	04	14
Missing records	01	00	00	00
Not applicable	00	00	07	179
Total no. With difficulty	26	119	79	28
% with impairment	8.7	40.0	26.3	23.1

**Fig 2:**
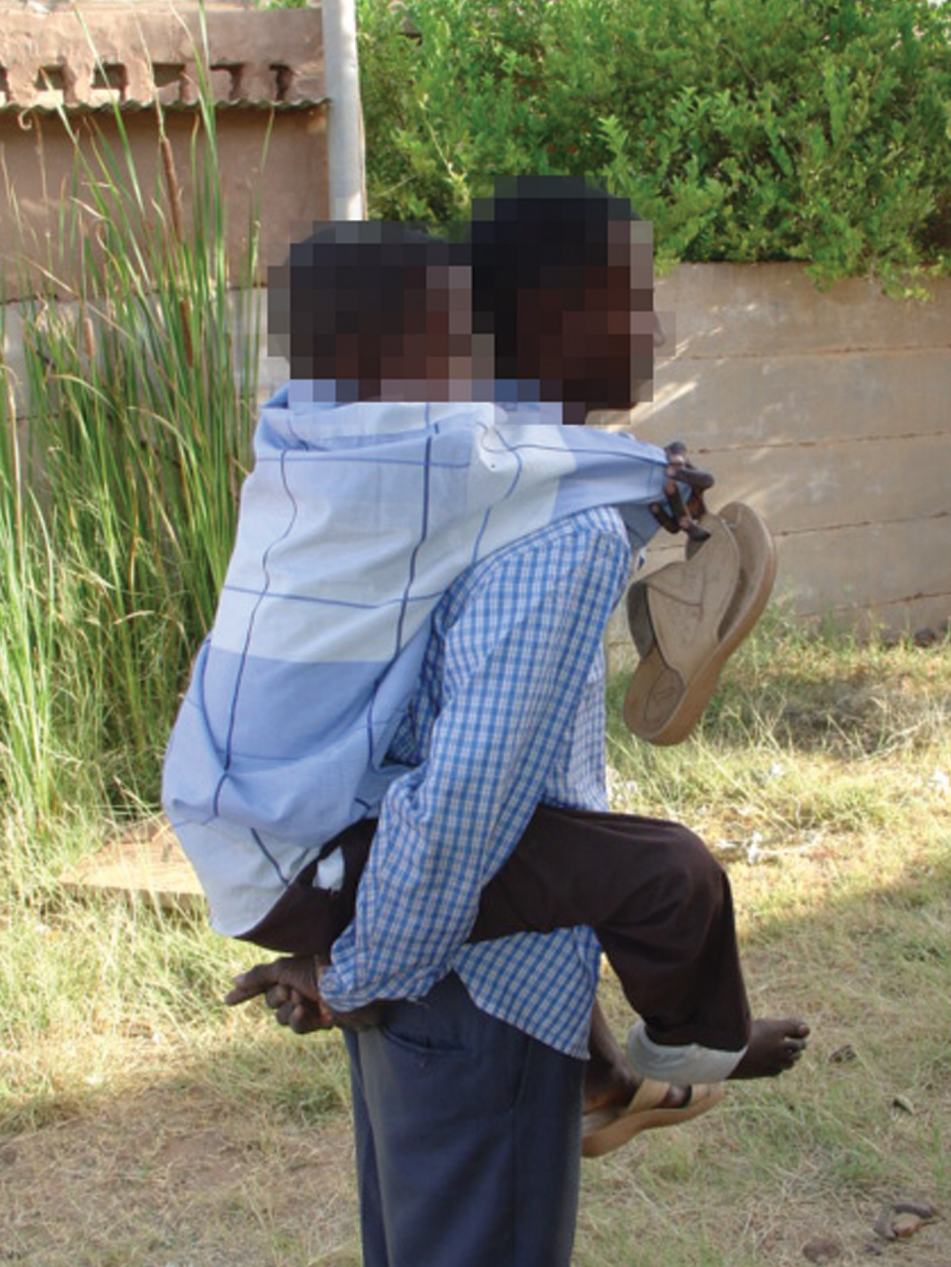
A patient carried consistently by his brother due to disability.

In self-care, the activities that predominantly require upper limb mobility are least affected by mycetoma and score lowest (eating, drinking, etc.; Table 5). Impaired ability to self-dress was reported by 112 patients (38%): mild impairment in 86 patients (29%), moderate in 22 patients (7%), severe in three patients (1%) and complete in one patient (0.3%). Impairment inability to dress oneself was statistically significantly correlated to the presence of pain at the mycetoma site (*p*<0.01); but not with advanced patient age or longer duration of the mycetoma. Proportionally, most impairment in self-care is marked as mild (dressing 76.8%, washing 71.2%, toileting 74.6%; Fig 2).

**Table 5:** Difficulties in self care.

Capacity	Washing	Toileting	Dressing	Eating	Drinking
No difficulty	225	242	187	291	292
Mild Difficulty	52	41	86	06	04
Moderate Difficulty	16	8	22	02	00
Severe Difficulty	02	02	03	00	00
Complete Inability	03	04	01	00	00
Missing	00	02	00	00	01
Not applicable	02	01	01	01	03
Total no. with difficulty	73	55	112	08	04
% with impairment	25.5	18.5	40.8	2.7	1.4

The study showed that 164 patients (55%) had difficulty in going about their daily life, (Table 6). A mild difficulty was reported by 63 patients (23%), 28 (9%) had moderate difficulty, 22 (7%) had severe difficulty, and 51 (17%) were completely unable to go about at least one aspect of their daily life because of the mycetoma. Many patients marked these sections as not applicable to them which was largely to do with male/female gender roles in domestic life. Generally speaking, in Sudan males are less likely to be involved in domestic life activities than females.

**Table 6:** Difficulties in domestic life activities.

Capacity	Shopping	Gathering Daily Necessities	Preparing Meals	Doing Housework
No difficulty	175	132	87	119
Mild Difficulty	23	12	10	18
Moderate Difficulty	07	05	04	12
Severe Difficulty	04	09	05	04
Complete Inability	15	20	07	09
Missing	00	01	01	01
Not applicable	76	121	186	137
Total no. with difficulty	49	46	26	43
% with impairment	21.9	25.8	23.0	26.5

Regarding interpersonal relationships, 57 (19%) patients reported difficulty, (Table 7). Mild difficulty was reported by 33 patients (11%), 15 patients (5%) reported moderate difficulty, six patients (2%) reported severe difficulty and three patients (1%) reported a complete inability to relate in the area of interpersonal interaction.

**Table 7:** Difficulties in interpersonal interactions and relationships.

Capacity	Informal Social Relationships	Family Relationships	Intimate Relationships
No difficulty	267	283	140
Mild Difficulty	15	13	5
Moderate Difficulty	10	2	3
Severe Difficulty	4	1	1
Complete Inability	3	0	0
Missing record	0	0	0
Not applicable	1	1	151
Total no. with some difficulty	32	16	9
% with impairment	10.7	5.4	6.0

Of the 300 interviewed patients, 37 were of school age (Table 8). Of these, 21 reported no difficulty at attending school while 16 reported present or previous difficulty. Twelve patients reported that mycetoma rendered them completely unable to continue their school education. Eighteen patients had the potential to gain higher education of whom seven experienced difficulty due to their mycetoma: four experienced mild difficulty, one experienced moderate difficulty, one experienced severe difficulty, and one was completely unable to continue their higher education because of their mycetoma. While the survey format did not allow us to collect information from all who reported that mycetoma had made continuing in school difficult or impossible, anecdotal comments to the data collectors identified mobility impairments that made walking to school difficult or impossible as the key barrier to continuing school attendance.

**Table 8:** Difficulties in attending education.

Capacity	School Education	Higher Education
No difficulty	21	11
Mild Difficulty	01	04
Moderate Difficulty	02	01
Severe Difficulty	01	01
Complete Inability	12	01
Missing	00	00
Not applicable	263	282
Total no. with some difficulty	16	07
% with impairment	44.4	38.9

Using the ICF definition of ‘economic status’ as “having economic resources from any source enough to ensure economic security in the present and the future” in the study, 126 patients (46.7%) reported a reduction in their ability to economically sustain themselves because of their mycetoma (Table 9). Mild difficulty was reported by 47 patients (17%) in their ability to be economically self-sufficient because of their mycetoma, 36 patients (13%) reported moderate difficulty, 19 patients (7%) reported severe difficulty, and 24 patients (9%) were completely economically non-self-sufficient because of their mycetoma.

**Table 9:** Difficulties in economic status.

Capacity	Remunerative Employment	Economic Self Sufficiency
No difficulty	104	143
Mild Difficulty	24	47
Moderate Difficulty	10	36
Severe Difficulty	06	19
Complete Inability	29	24
Missing	00	01
Not applicable	127	30
Total no. with some difficulty	69	126
No. with impairment %	39.9	46.7

Specifically, 69 patients (40%) out of a relevant 173 patients found difficulty in their remunerative employment: 24 patients (14%) experienced mild difficulty, 10 patients (6%) moderate, six patients (4%) severe and 29 patients (17%) were completely unable to work for remunerative gain because of their mycetoma. There were 127 non-applicable individuals, who were not in work previously.

Impairment in remunerative employment was significantly correlated with the presence of pain (*p*<0.05), impairment in walking (*p*<0.01) and impairment in economic self-sufficiency (*p*<0.01). Impairment in economic self-sufficiency was also significantly correlated with the presence of pain (*p*<0.01) and impairment in walking (*p*<0.01) but also advanced age (*p*<0.01). Neither was correlated with duration of the mycetoma.

**Fig 3:**
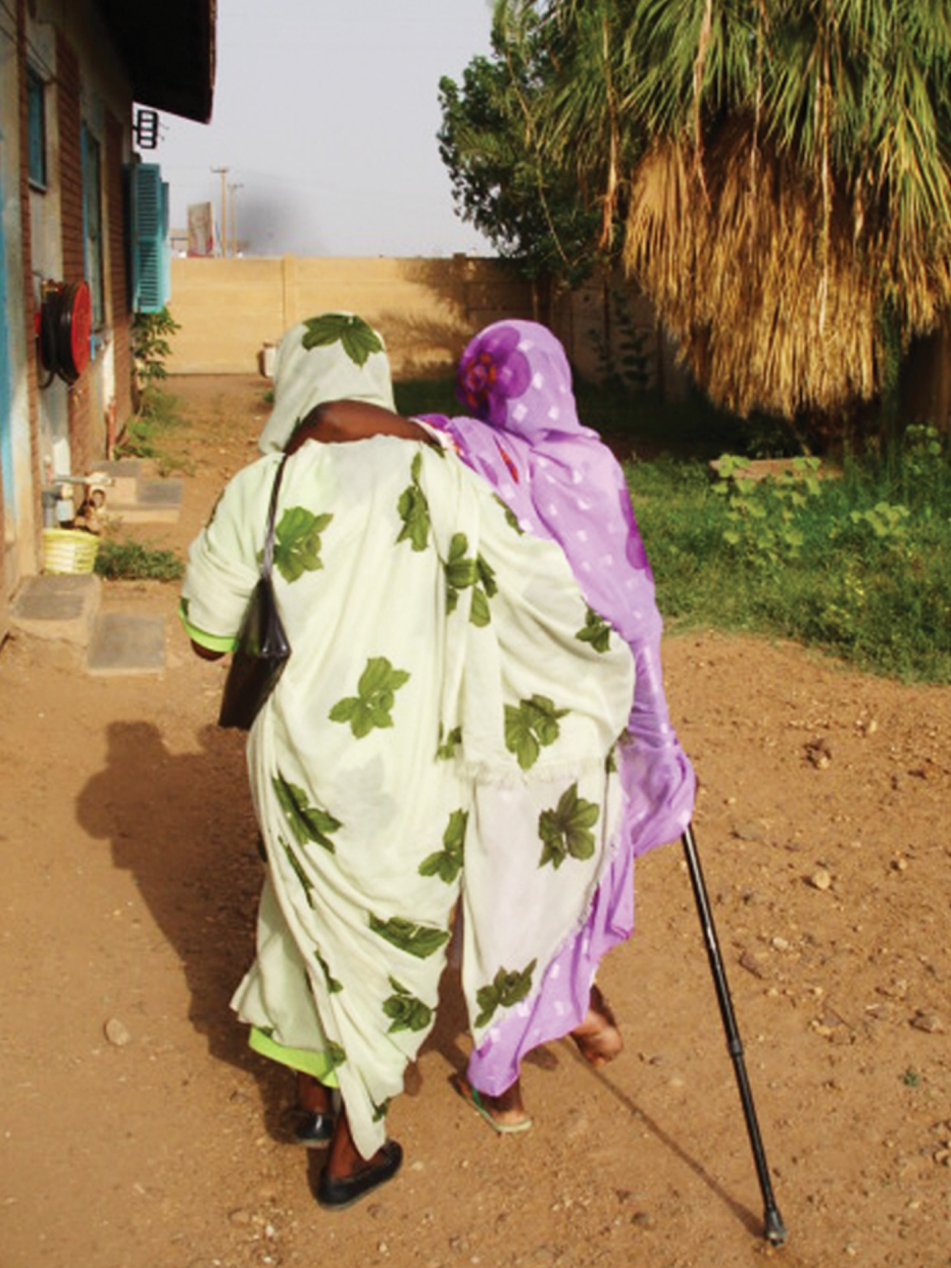
A lady supported by her sister to attend social event.

Of the 296 patients who reported that participation in a range of community activities were important to them, 63 (21.3%) experienced difficulty in participation because of the mycetoma, with 20 patients (7%) reporting mild, 22 patients (8%) moderate, and six patients (2%) severe limitations (Table 10). An additional 15 patients (5%) reported that they were completely unable to participate in community events or ceremonies. Of the 216 patients to whom recreation or leisure was important, 21 patients (9.7%) experienced difficulty in participation. Of the 107 patients to whom sports were relevant, 68 patients (63.6%) experienced difficulty, of whom four patients (4%) experienced mild difficulty, six patients (6%) moderate difficulty, seven patients (7%) severe, and 51 (48%) reported being completely unable to participate in sports because of their mycetoma. Of this study population, 54 patients out of 290 (19%) experienced difficulty in socialising, whilst 71 patients out of 295 (24.1%) experienced difficulty in participating in religious activities (Fig 3).

**Table 10:** Impairments in community, social and civic life.

Capacity	Ceremonies	Recreation & Leisure	Sports	Socialising	Religion
No difficulty	233	195	39	236	224
Mild Difficulty	20	09	04	26	39
Moderate Difficulty	22	03	06	10	24
Severe Difficulty	06	02	07	8	3
Complete Inability	15	07	51	10	05
Missing	00	0	0	0	00
Not applicable	04	84	193	10	05
Total no. with some difficulty	63	21	68	54	71
No. with impairment %	21.3	9.7	63.6	18.6	24.1

## Discussion

There is a general paucity of literature investigating the nexus of neglected tropical diseases and disability, though existing work supports our main findings. A study addressing leprosy-related disability highlights the disease’s economic impact and association with extreme poverty. It also shows that people with a leprosy-related disability achieved fewer individual and family related objectives without interventions recognising their disability [32]. Work in malaria and tuberculosis also shows the importance of qualitative approaches in elucidating the nature of the disease-related disability, its interaction with poverty and related barriers to care [33, 34].

Our study is the first which measures and characterises mycetoma-related disability. It also clearly links mycetoma-related disability with a negative impact on economic livelihood. Overall, 83.7% of the studied patients report disability in one or more of the 33 ICF domains examined, with 60.3% reporting at least moderate disability in one or more domain. Further analysis reveals areas of differential impact along the lines of gender and age, alongside specific vulnerabilities common to all those with mycetoma, including pain, impaired mobility and stigma.

Four times as many males are diagnosed with this disease as females, suggesting variations either in incidence patterns or in societal responses, and hinting at gender-based vulnerabilities requiring further research. There may be true gender variation in disease incidence, relating to increased likelihood of traumatic injury allowing pathogenic inoculation, or to a hormonal influence on disease progression in males [35]. An alternative explanation is that women may be less likely to seek or receive medical care due to stigma, financial or ‘gender-role’ considerations, leading to reporting bias. This alternative is supported by a village level survey which showed that gender and age distributions were more evenly distributed than those measured at the tertiary level [15]. Indeed in endemic areas, females may in fact have similar risks of exposure as men, due to extensive outdoor activities. Either explanation hints at a particular vulnerability rooted in gender.

There are two striking age-related impacts. Firstly, older adults bear a greater burden of disability overall than younger patients or students, suggesting that symptoms may correlate with both disease duration, and therefore disease progression, and with physical activity and pain. Secondly, there is a strong effect on educational attendance in the school-age population where 44.4% of students with mycetoma experience difficulty in attending primary school, and 38.9% struggle to attend higher education. Our study did not formally assess this effect further, but physical inability to walk to school was mentioned by several interviewees as an underlying cause. For school-age children with little or no access to transport to school facilities, this would represent a strong barrier to accessing education. Illness and disease have also been shown to negatively impact a child’s learning and school time in less developed settings [36]. The combined effects of decreased access and poorer quality of learning when in lessons are likely to result in unfulfilled potential and loss of future opportunities.

Three further mycetoma-related effects are worth discussing for their creation of particular vulnerabilities common to all individuals with the disease. These include pain, limited mobility and stigma. Mycetoma is typically characterised as a painless disease, although there are reports of local pain at the mycetoma site and that is frequently due to secondary bacterial infection [37, 38, 39]. However, in this study over a third of respondents (103 patients, 34.3%) reported local pain, higher than has been previously reported [2, 4, 20]. Whilst most cases were mild, infrequent and tolerable, this challenges the idea that mycetoma is generally painless. It is likely to result in vulnerabilities correlating with poorer development and achievement outcomes including school attendance, academic performance and the ability to perform physical tasks, both domestic and professional.

Walking itself receives high impact scores with 119 patients (40.0%) complaining of restriction in their ability to walk without assistance. The assessment questions point towards impairment being produced by the mycetoma itself, rather than other co-morbidities or age-associated frailties. In general, co-morbidities are rare in mycetoma as most of the patients by number are young adults and children (though notably those with the most disabling consequences are the elder patients). The associations between a sedentary lifestyle and non-communicable diseases are well-known however and those with mycetoma-related physical restriction are likely to be at increased risk for them.

A substantial number of respondents reported reduced participation in education, community, religious, social and civic life. This could be partly a result of stigma and social isolation, either because of specific beliefs about the disease e.g. contagion, linkage of physical and spiritual malady, or due to broader prejudice against individuals with a disability.

However, less than 20% of respondents reported difficulties in socialising (18.6%), with just 7% reporting difficulties in interpersonal relationships overall. Furthermore, relatively few patients had impairments in such bodily functions as energy and drive (26%), emotional function (25%) or body image impairment (27.5%). These findings might be explained by the localised, slowly progressive nature of the disease, together with the rarity of cosmetically ‘significant’ areas, such as the face, being affected.

It is also interesting to note that few patients had impairment in informal social- (11%), family- (5%) or intimate- (6%) relationships. This may be a finding particular to the study setting, partly related to the strong concept of the extended families in the Sudan, where patients receive support and care from many family members even if they are not close relatives.

Overall these findings suggest that in the context of Sudan reduced participation in aspects of public life may be influenced as much by physical issues as by social stigma. Further work is needed to explore this complex area.

The most common limitation, by a number of people affected, was in economic self-sufficiency (126 affected, 46.8% of applicable patients). Self-sufficiency here was defined as having enough economic resources from any source to ensure economic security in the present and the future [30]. A component of this is likely to be disease-related inability to work. Indeed 40% of those who would otherwise be in work were partially or completely unable to engage in remunerative employment because of their mycetoma. Our results do not explore the exact nature of this loss in economic self-sufficiency, however we know that even where treatment is available free of charge, such as that provided at the MRC, indirect healthcare costs (e.g. cost of transport, loss of paid work) are consistently a significant burden on household economies [40]. An emerging body of research evidence also shows that individuals living with a disability, and households with disabled members, face increased costs compared to those without, e.g. for medical care, transportation, loss of paid work both by the affected individual and by those who remain at home to provide care [41,42].

In this study, there were four main limitations. Firstly, data collection was based on the ICF Model, rather than the Washington Group (WG) Questions on Disability Statistics [43]. This was because the project was initiated and data was collected prior to awareness of the WG methodology by field research staff. There is some overlap, since the WG questions are based upon the ICF Model, however the WG methodology captures dimensions of individual functioning more precisely and should be the basis of future work. Nevertheless, we believe our data remains valid, relevant and yields new insight into mycetoma-related disability. Secondly, data collectors were recent medical graduates without backgrounds in research or disability. Therefore extensive training and support was given to minimise the risk of decreased accuracy or reliability of results. Third, our data is predominantly quantitative, save for anecdotes reported to data collectors. A qualitative component in further research could add further valuable insight into how individuals and societies are affected. And finally, although this was not the purpose of the study, we did not record whether patients had actinomycetoma or eumycetoma and are unable to perform a subgroup analysis of these groups to look for differences in disability.

In conclusion, this is the first study to outline the burden of disability caused by mycetoma. It shows clear evidence of mycetoma-related impact on individuals' body function, mobility, and ability to self-care, with significant ramifications for domestic life, interpersonal relationships, educational attainment, economic status, and civic engagement. There is sufficient evidence to support specific interventions aiming to mitigate and adapt to the disabling consequences of mycetoma. However, better detection and disease education are also important for preventing infection and the development of mycetoma-related disability.

Clinically, effective pain management should be routinely integrated into mycetoma services, particularly for those groups identified as higher risk such as farmers and older adults. Different analgesic agents can be evaluated for efficacy against mycetoma-related pain. More definitive treatment should include the elimination of the secondary bacterial infection by appropriate anti-pathogenic agents and repeated lesion debridement. Further addition of physiotherapy services could begin to address limitations of mobility and function. Simple walking aids could be a starting point followed by in-house physiotherapy services. They could also provide a starting point for reducing risks associated with sedentary lifestyles.

In education, services could include assessment of a student’s mycetoma-related disability using a pragmatic screening tool based on our results. This would inform adaptations in educational provision, including greater local awareness and consideration of learning aids or adaptations that allow distance learning, participation in physical and sporting activities, in modifying transport to and from school as well as mitigating stigma in educational settings.

This study shows evidence of impaired revenue generation at an individual level and a negative impact on economic self-sufficiency and household income. While this establishes that diseases such as mycetoma have significant development and public health implications, further research is needed to quantify its financial effects at the individual, household and societal levels. This includes effects on revenue generation, career opportunities and health-related costs, including the reallocation of disposable assets.

Above all, our data emphasise that the multi-faceted, long-term disabling consequences of NTDs, such as mycetoma, must not be overlooked. Their effects are likely to be felt at the level of individuals, families and communities, across multiple social and economic dimensions. Importantly, such effects are likely to be modifiable and therefore must be considered and addressed in future policy and programming.
